# Psychometric Features of the Edinburgh Postnatal Depression Scale Among Malaysian Women: A Cross-Sectional Study

**DOI:** 10.7759/cureus.34822

**Published:** 2023-02-09

**Authors:** Adam Bied, Susan Njuguna, Nurul Husna Mohd Shukri, Zurina Zainudin

**Affiliations:** 1 Department of Psychiatry and Behavioral Sciences, ABC Medical, Reading, USA; 2 Laboratory of Youth and Community Well, Institute for Social Science Studies, Universiti Putra Malaysia, Serdang, MYS; 3 Faculty of Medicine and Health Sciences, Department of Nutrition, Universiti Putra Malaysia, Serdang, MYS; 4 Faculty of Medicine and Health Sciences, Department of Pediatrics, Universiti Putra Malaysia, Serdang, MYS

**Keywords:** consultation liaison psychiatry, public psychiatry, screening program, edinburgh postnatal depression scale, postpartum depression

## Abstract

Background

The Edinburgh Postnatal Depression Scale (EPDS) is a 10-item questionnaire developed to identify women at risk for postpartum depression (PD). EPDS symptom patterns appear to vary by nation. The EPDS items and their correlation with affirmative EPDS screens have thus far been minimally studied among Malaysians. Using positive predictive value (PPV) and negative predictive value (NPV), this study aims to evaluate the EPDS-based psychometric features of individual answer items.

Methodology

A cross-section of postpartum women receiving care at two tertiary care facilities in Malaysia underwent screening assessments in the course of receiving treatment. EPDS was employed with an aggregate cutoff score (≥12). EPDS items were assessed as predictors of abnormal EPDS screens.

Results

A total of 219 participants were screened, among which 66 were positive on the EPDS. EPDS item responses were collected (item responses 0-3: PPV = 0.07-0.78 and NPV = 0.93-0.22). A negative response to any item strongly predicted a negative EPDS screen (item entry = 0 and NPV = 0.93). Affirmative responses on items 8, 9, and 10 were particularly strong predictors of abnormal EPDS scores, while negative responses to items 3, 5, and 7 were strong predictors of negative EPDS scores. A substantial NPV for any item (response 0 and NPV items 1-10 = 0.93) and a moderate PPV for any affirmative response (responses 1-3, PPV items 1-10 = 0.60) were observed.

Conclusions

This is one of the few studies to examine the EPDS item responses among Malaysian women. The results suggest that depression remains prevalent in this postpartum population. Our findings reveal a robust NPV for any negative response to the individual items of the scale, a moderate PPV for any affirmative response, and a particularly robust validity for specific EPDS items. Physical complaints, rather than feelings of sadness, figure prominently in this population, suggesting a tendency among Malaysian women toward somatization.

## Introduction

Postpartum depression (PD) is a common disorder, affecting approximately 14% of all women following childbirth [1]. The Edinburgh Postnatal Depression Scale (EPDS) has become a leading choice for identifying women at risk for the diagnosis [2]. Prior literature has shown that EPDS symptom patterns vary by nation [3]. Clinicians have come to commonly use this screen in clinical practice and various professional associations have proffered guidelines on its use. The EPDS is a 10-item questionnaire, which has been validated in different populations [4,5,6]. Despite this, considerable debate persists regarding the optimal means of using this instrument. Some authors have advocated for one- or two-factor scoring, others for different cutoffs, and others still for using the scale at different points in treatment. The scale’s validity in various languages has also been the subject of study, with efforts among clinicians aimed at validating certain translated forms of the questionnaire. The individual symptoms, presenting complaints, and areas of dysfunction have also varied considerably across different populations. The manifestations of PD, it has been argued, differ along a cultural spectrum with different EPDS symptom patterns observed in various nations [3]. Thus far, a few studies have evaluated this subject among Malaysian women. Assessing EPDS symptom patterns may be beneficial in understanding PD and EPDS reports among this population. Here, we assess the predictive features, both positive and negative (positive predictive value [PPV] and negative predictive value [NPV]), of EPDS items relative to that of aggregate EPDS scores.

## Materials and methods

This is a continuation of a previously published cross-sectional study of women receiving care at two hospitals in the Klang Valley of Malaysia [7]. Participants comprising women who had given birth six or fewer months prior completed an EPDS. Additional inclusion requirements were the completion of written consent, completion of the entire EPDS, and proficiency in either Malay or English. The participants were offered either the Malay or English version of the EPDS at the time of assessment. The Medical Research Ethics Committee (MREC) of Malaysia approved this study (MREC approval was granted in January 2020 under ID NMRR-19-3216-51651). The predictive features of the individual item responses for an overall positive EPDS screen, items 1-10, and responses 0-3 were analyzed with standardized cutoffs (≥12). Cutoffs were established by prior studies validating the Malaysian variants of EPDS [7]. Participants' characteristics, including age, ethnicity, education level, employment status, parity, and prematurity, were solicited. NPV and PPV were calculated to evaluate the psychometric features of the EPDS individual answer items.

## Results

Among 331 potential participants approached, 294 met the inclusion criteria, of which 219 completed the study assessment. Among study completers, 66 experienced positive EPDS scores. Participants' demographics, including age, ethnicity, education, employment, marital status, parity, prematurity, and income, were solicited (Table 1).

**Table 1:** Sociodemographic characteristics of the study participants (n = 219). ^a^USD 1 = RM 4.45 (conversion rate in August 2022). USD, United States dollar; RM, Ringgit Malaysia; SD, standard deviation

Characteristics	n	%	Mean	SD
Age (years)	219	100	31.6	4.9
Ethnicity				
Malay	198	90.5		
Chinese	11	5		
Indian	6	2.7		
Bumiputera	4	1.8		
Marital status				
Married	216	98.6		
Education level				
Primary/secondary education	57	26		
Pre-university	78	35.6		
Tertiary education	84	38.4		
Occupation				
Employed	165	75.3		
Unemployed	54	24.7		
Household income^a^				
Low (<3,800)	100	46.7		
Middle (RM 3,800-8,300)	82	38.3		
High (>RM 8,300)	32	15		

The predictive validity, both positive and negative, for each item, including the respective item responses 0, 1, 2, and 3, were calculated relative to that of the EPDS screen. Mean EPDS scores were 9.37 for all the participants, 16.12 for those screening positive, and 6.46 for those screening negative (Table 2).

**Table 2:** Predictive validity of EPDS item metrics (PPV and NPV). EPDS, Edinburgh Postnatal Depression Scale; PPV, positive predictive value; NPV, negative predictive value; Q1 to Q10, Questions 1 to 10

Item number	Item question	PPV				NPV			
		Score 0	Score 1	Score 2	Score 3	Score 0	Score 1	Score 2	Score 3
Item Q1	I have been able to laugh and see the funny side of things.	0.13	0.64	0.60	0.54	0.87	0.36	0.40	0.46
Item Q2	I have looked forward with enjoyment to things.	0.16	0.54	0.63	0.50	0.84	0.46	0.38	0.50
Item Q3	I have blamed myself unnecessarily when things went wrong.	0.00	0.24	0.39	0.70	1.00	0.76	0.61	0.30
Item Q4	I have been anxious or worried for no good reason.	0.08	0.18	0.66	0.67	0.92	0.82	0.34	0.33
Item Q5	I have felt scared or panicky for no very good reason.	0.00	0.18	0.72	0.82	1.00	0.82	0.28	0.18
Item Q6	Things have been getting on top of me.	0.04	0.18	0.95	0.71	0.96	0.82	0.38	0.29
Item Q7	I have been so unhappy that I have had difficulty sleeping.	0.04	0.17	0.54	0.88	0.96	0.83	0.46	0.13
Item Q8	I have felt sad or miserable.	0.05	0.25	0.96	1.00	0.95	0.75	0.04	0.00
Item Q9	I have been so unhappy that I have been crying.	0.04	0.20	0.82	0.94	0.96	0.80	0.18	0.06
Item Q10	The thought of harming myself has occurred to me.	0.20	0.65	0.88	1.00	0.80	0.35	0.12	0.00
Mean item all	EPDS questionnaire mean score	0.07	0.32	0.71	0.78	0.93	0.68	0.32	0.22

## Discussion

This is one of the few studies assessing EPDS item validity among Malaysian women. Our findings comprising 219 participants and 8,760 data entries reveal useful information about the EPDS among Malaysians. Participants' demographics mirrored the literature on this population [8]. In reviewing the findings, any negative item response strongly predicts a negative EPDS screen (NPV 0.93). An affirmative response, however, to question 1, about being *able to laugh and see the funny side of things*, and question 2, about looking *forward with enjoyment to things*, poorly predicted a positive EPDS score (PPV response 3 = 0.54 and 0.50). Affirmative responses to item 10, about thoughts of suicide, incompletely predicted a positive EPDS screen (PPV, responses 1, 2, and 3 = 0.65, 0.88, and 1.0, respectively). Item 8, relating to being *sad and miserable*, and item 9, relating to being *unhappy* and *crying*, were strong predictors of positive screens (item 8 PPV responses 1, 2, and 3 = 0.25, 0.96, and 1.00, respectively; item 9 PPV responses 1, 2, and 3 = 0.20, 0.82, and 0.94, respectively).

The authors proffer two explanations for these findings. The first is that some symptoms may be shared with an alternative mental health diagnosis. Postpartum blues, or *baby blues*, is a mild mood variant, which has been hypothesized to occur in as much as four-fifths of all women following birth [9]. Birth-related post-traumatic stress disorder (PTSD) is a newly elucidated diagnosis thought to manifest in some postpartum populations [10]. Epidemiology studies have also suggested that postpartum psychosis may occur in as much as one in 500 women [11], and recent research has suggested some women experience a variant of bipolarity emerging during the postpartum period [12]. Collectively, one may suspect that some, or perhaps even significant numbers, of the study participants experienced one or more of these diagnoses, or other psychiatric ailments not reviewed here, and for this reason, scored affirmatively on one or more items of the EPDS while also failing to experience either an affirmative screen or the PD diagnosis. Research assessing this may be elucidated by conducting a more comprehensive psychiatric assessment concurrently with administering the EPDS or conversely employing such assessment in scenarios where the screen is negative but items on it are affirmative.

The second explanation we offer pertains to symptom interpretation. One may reason that participants might communicate a psychiatric complaint when voicing a physical worry. The manifestations of PD have been shown to vary by region, with sadness being a leading complaint in industrialized nations and somatic symptoms in less developed nations [3]. Consistent with this, item 7, about sleep, was only somewhat less predictive (PPV response 3: 0.88) than item 10, about suicidality (PPV response 3: 1.0). Moreover, items 1, 2, and 3, which relate to being *able to laugh and see the funny side of things*, looking *forward with enjoyment to things*, and blaming oneself *unnecessarily when things went wrong*, were relatively weak predictors of EPDS scores, despite displaying favorable face validity. Two systematic reviews, one published in 2016 [13] and another in 2021 [14], revealed poor validity for EPDS items, particularly for items about guilt and self-blame, among some low-income and indigenous cultures. Our findings appear to support such findings among the population studied and raise some doubts about the optimal interpretation of negative responses to certain EPDS items.

Given these findings, clinicians may wish to be mindful of several features of EPDS in this population. Any item answered negatively strongly predicted a negative EPDS screen, while any positive response varied in its prediction of a positive one (item 7, response 1 = 0.27; items 8, 9, and 10 response 3 = 1.0, 1.0, and 1.0). As such, some clinicians may wish to consider either different EPDS cutoffs, differential weighting of EPDS items, or a combination of these modifications. Additionally, employing assessment protocols that include additional items such as questions about alternative behavioral health diagnoses, physical symptoms explainable by both psychiatric and general medical illnesses, and those about patients' perceptions of mental health may be desirable. Barring this, clinicians may wish to introduce such content into interviews as a means of developing their differential and more appropriately responding to the needs of this population.

The authors contend that the EPDS is a practical instrument for screening postpartum women in Malaysia and that affirmative screens are far from rare in this population. More relevantly, our findings suggest that symptom distributions vary significantly in this population when compared to populations studied elsewhere [13]. Prior studies have proffered the EPDS to be a valid predictor of PD as well as an instrument with an optimized cutoff of 12 among Malaysians [13,15,16]. Alternatively, our findings suggest that this population displays a unique clustering of symptoms among those positive on the screen. Clinicians may wish to employ a culturally adaptive understanding in this population, particularly when developing diagnostic impressions, counseling clients affirmative for EPDS, and assessing screening protocols for PD.

Limitations

Without conducting a comprehensive diagnostic interview, it is impossible to determine who among the study participants experienced PD. Our review assesses only the predictive features of the individual EPDS items relative to that of the EPDS screen results. Subsequent studies may wish to integrate such assessment into their study design, aware that the EPDS's predictive value for the PD is incomplete. A more optimal approach may be to compare EPDS items to that of the PD itself, aware that there may be incomplete parity between the EPDS results and the findings of a comprehensive psychiatric interview. Another approach may be to assess item response relative to psychiatric, pregnancy, or pediatric outcomes. Regardless, our study did not assess either, and hence, some hesitation may be expected when making firm conclusions about any item response. Incomplete participant enrollment remains another limitation of our study. Among 294 patients eligible for study enrollment, only 219, or 74.5%, completed the study assessment, and hence, our data catchment may be impacted by such non-enrollment. The authors offer two responses - first, in clinical settings, some subsets of patients may refuse to complete an EPDS questionnaire, and we speculate that such non-enrollees may be comparable to those in our study. Second, literature validating the EPDS has historically done so with similar degrees of non-enrollment, and hence, our findings may be true of the broader literature on the subject. The authors note that the sample size studied, 219, of which 66 screened affirmatively on the EPDS, may be viewed by some as small. The authors concede that while a larger sample size would be preferable, this remains one of the larger studies thus far conducted on this subject and may contribute to existing data on this population. Finally, the findings were drawn from the population studied. It is impossible to make assessments about the broader Malaysian population or about individuals in the Klang Valley, the location of our study, who receive treatment in other care settings. Barring such data, the authors would content that their findings may be the best available on the subject and, hence, the most generalizable presently available for Malaysians.

## Conclusions

This is the first study to examine EPDS item validity among Malaysian women. The results suggest that PD remains prevalent in this postpartum population. Our findings show a robust NPV for any negative response to the individual items of the scale, a moderate PPV for any affirmative response, and a particularly robust PPV for items 8, 9, and 10. Physical complaints, rather than feelings of sadness, figure notably in this population suggesting a tendency among Malaysian women towards somatization. Further research is warranted, particularly for items that despite inclusion on the scale reveal incomplete PPV and those which display robust face validity but have less substantial predictive value. In the absence of such literature, clinicians may wish to assess alternate behavioral health diagnoses, medical causes of positive EPDS symptoms, and patient perceptions of mental illness.

## References

[1] Frieder A, Fersh M, Hainline R, Deligiannidis KM (2019). Pharmacotherapy of postpartum depression: current approaches and novel drug development. CNS Drugs.

[2] Cox JL, Holden JM, Sagovsky R (1987). Detection of postnatal depression. Development of the 10-item Edinburgh Postnatal Depression Scale. Br J Psychiatry.

[3] Zubaran C, Schumacher M, Roxo MR, Foresti K (2010). Screening tools for postpartum depression: validity and cultural dimensions. Afr J Psychiatry (Johannesbg).

[4] Bergant AM, Nguyen T, Heim K, Ulmer H, Dapunt O (1998). Deutschsprachige fassung und validierung der Edinburgh postnatal depression scale [German language version and validation of the Edinburgh postnatal depression scale]. Dtsch Med Wochenschr.

[5] Vivilaki VG, Dafermos V, Kogevinas M, Bitsios P, Lionis C (2009). The Edinburgh Postnatal Depression Scale: translation and validation for a Greek sample. BMC Public Health.

[6] Mohamad Yusuff AS, Tang L, Binns CW, Lee AH (2015). Prevalence and risk factors for postnatal depression in Sabah, Malaysia: a cohort study. Women Birth.

[7] Mohd Shukri NH, Senjaya O, Zainudin Z, Mohamed M, Syed Abdullah FI (2022). The associations of breastfeeding and postnatal experiences with postpartum depression among mothers of hospitalized infants in tertiary hospitals. Cureus.

[8] Nasreen HE, Pasi HB, Rifin SM, Aris MA, Rahman JA, Rus RM, Edhborg M (2019). Impact of maternal antepartum depressive and anxiety symptoms on birth outcomes and mode of delivery: a prospective cohort study in east and west coasts of Malaysia. BMC Pregnancy Childbirth.

[9] Bobo WV, Yawn BP (2014). Concise review for physicians and other clinicians: postpartum depression. Mayo Clin Proc.

[10] Ayers S, Wright DB, Thornton A (2018). Development of a measure of postpartum PTSD: the city birth trauma scale. Front Psychiatry.

[11] Rodriguez-Cabezas L, Clark C (2018). Psychiatric emergencies in pregnancy and postpartum. Clin Obstet Gynecol.

[12] Hutner LA, Catapano LA, Nagle-Yang SM, Williams KE, Osborne LM (2021). Textbook of Women's Reproductive Mental Health. https://books.google.com.my/books.

[13] Shrestha SD, Pradhan R, Tran TD, Gualano RC, Fisher JR (2016). Reliability and validity of the Edinburgh Postnatal Depression Scale (EPDS) for detecting perinatal common mental disorders (PCMDs) among women in low-and lower-middle-income countries: a systematic review. BMC Pregnancy Childbirth.

[14] Chan AW, Reid C, Skeffington P, Marriott R (2021). A systematic review of EPDS cultural suitability with Indigenous mothers: a global perspective. Arch Womens Ment Health.

[15] Levis B, Negeri Z, Sun Y, Benedetti A, Thombs BD (2020). Accuracy of the Edinburgh Postnatal Depression Scale (EPDS) for screening to detect major depression among pregnant and postpartum women: systematic review and meta-analysis of individual participant data. BMJ.

[16] Park SH, Kim JI (2022). Predictive validity of the Edinburgh postnatal depression scale and other tools for screening depression in pregnant and postpartum women: a systematic review and meta-analysis. Arch Gynecol Obstet.

